# Differential Expression and Sex Chromosome Association of CHD3/4 and CHD5 during Spermatogenesis

**DOI:** 10.1371/journal.pone.0098203

**Published:** 2014-05-21

**Authors:** Judith W. Bergs, Nina Neuendorff, Godfried van der Heijden, Evelyne Wassenaar, Peter Rexin, Hans-Peter Elsässer, Roland Moll, Willy M. Baarends, Alexander Brehm

**Affiliations:** 1 Institute for Molecular Biology and Tumor Research (IMT), Philipps Universität Marburg, Marburg, Germany; 2 Department of Reproduction and Development, Erasmus MC-University Medical Center, Rotterdam, The Netherlands; 3 Institut für Pathologie, Philipps Universität Marburg, Marburg, Germany; 4 Institut für Zytobiologie und Zytopathologie, Philipps Universität Marburg, Marburg, Germany; University of Nevada School of Medicine, United States of America

## Abstract

ATP-dependent nucleosome remodelers of the CHD family play important roles in chromatin regulation during development and differentiation. The ubiquitously expressed CHD3 and CHD4 proteins are essential for stem cell function and serve to orchestrate gene expression in different developmental settings. By contrast, the closely related CHD5 is predominantly expressed in neural tissue and its role is believed to be restricted to neural differentiation. Indeed, loss of CHD5 contributes to neuroblastoma. In this study, we first demonstrate that CHD5 is a nucleosome-stimulated ATPase. We then compare CHD3/4 and CHD5 expression in mouse brain and show that CHD5 expression is restricted to a subset of cortical and hippocampal neurons whereas CHD3/4 expression is more widespread. We also uncover high levels of CHD5 expression in testis. CHD5 is transiently expressed in differentiating germ cells. Expression is first detected in nuclei of post-meiotic round spermatids, reaches a maximum in stage VIII spermatids and then falls to undetectable levels in stage IX spermatids. Surprisingly, CHD3/4 and CHD5 show complementary expression patterns during spermatogenesis with CHD3/4 levels progressively decreasing as CHD5 expression increases. In spermatocytes, CHD3/4 localizes to the pseudoautosomal region, the X centromeric region and then spreads into the XY body chromatin. In postmeiotic cells, CHD5 colocalises with macroH2A1.2 in association with centromeres and part of the Y chromosome. The subnuclear localisations of CHD4 and CHD5 suggest specific roles in regulation of sex chromosome chromatin and pericentromeric chromatin structure prior to the histone-protamine switch.

## Introduction

ATP-dependent nucleosome remodelers are important regulators of the epigenome. They utilise energy derived from ATP hydrolysis to modulate histone:DNA interactions resulting in nucleosome repositioning, nucleosome assembly and disassemby or histone variant exchange. Nucleosome remodelers affect a variety of fundamental biological processes including gene transcription, DNA repair, DNA replication, chromosome cohesion, stem cell maintenance and differentiation. Mutation or misregulation of nucleosome remodelers can cause a variety of diseases ranging from developmental disorders to cancer [1].

**Figure 1:**
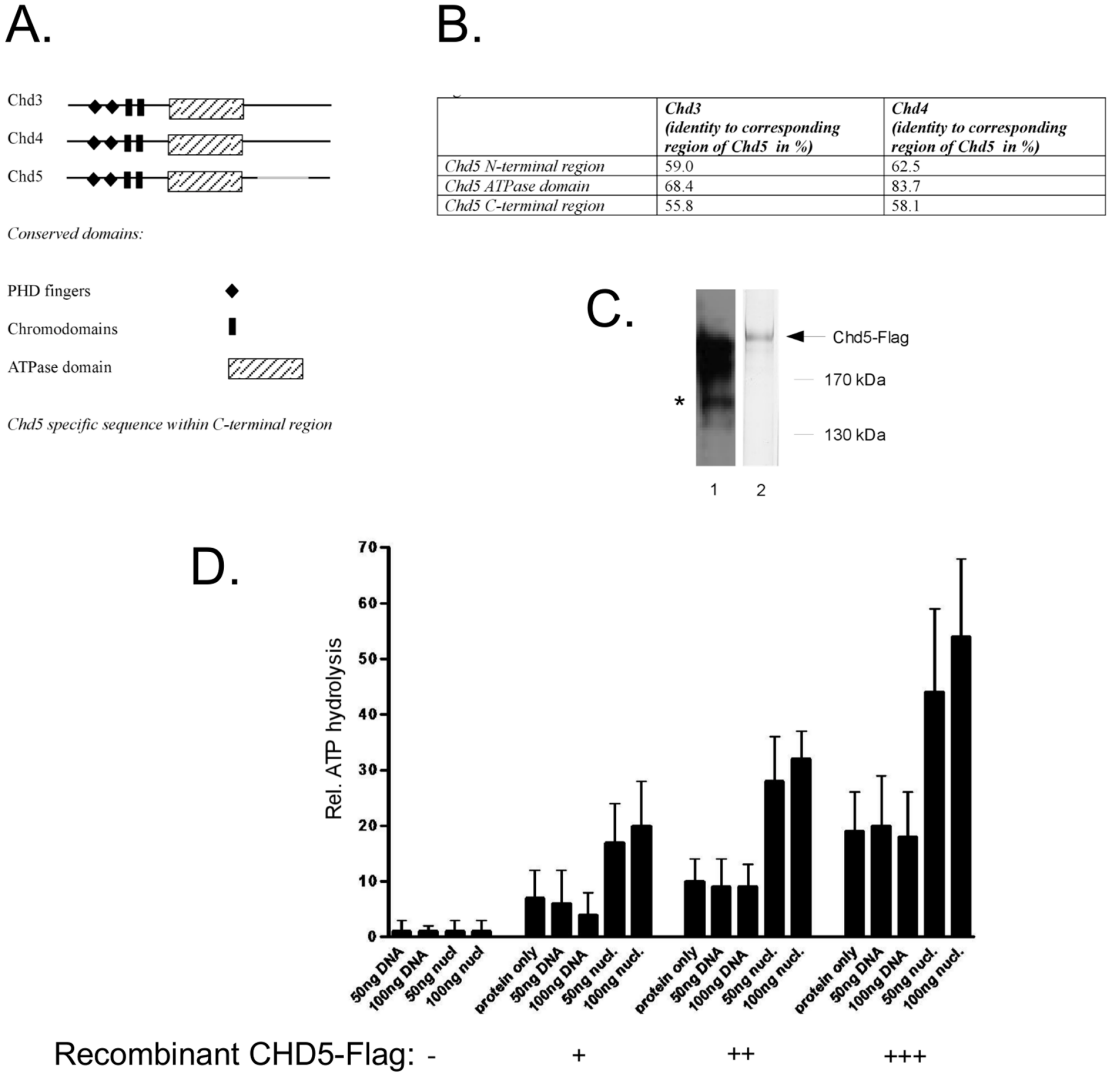
CHD5 is a nucleosome stimulated ATPase. (A) Schematic drawing of CHD3, CHD4 and CHD5 proteins showing conserved domains and the CHD5 specific sequence. (B) Table showing sequence identity between different domains of CHD3, CHD4 and CHD5. (C) Expression of recombinant CHD5. Recombinant CHD5-Flag was purified from extracts of baculovirus-infected Sf9 cells. Extracts were incubated with anti-Flag affinity resin, washed and eluted with an excess of Flag peptide. Purified protein was seperated by SDS-PAGE followed by Western blotting with CHD5 antibody (lane 1) or silver staining (lane 2). Asterisk indicates degradation products with intact anti-CHD5 epitope. (D) CHD5 is a nucleosome-stimulated ATPase. Purified recombinant CHD5-Flag was subjected to ATPase assay as described in Materials and Methods. Different amounts of recombinant human CHD5-Flag, naked DNA or *in vitro* assembled nucleosomes were used as indicated at the bottom of the panel. Reaction products were separated by thin layer chromatography and the amount of released ^32^P_i_ was quantitated. The percentage of ATP hydrolysis was calculated for each reaction. The percentage of ATP hydrolysis of control reactions (without enzyme) was set to one. Error bars depict the standard deviation of 3 independent experiments.

ATP-dependent nucleosome remodelers share a conserved SNF2-type ATPase/helicase domain [2]. They are grouped into distinct subfamilies based on the presence of additional domains. The chromodomain-helicase-DNA-binding (CHD) family of nucleosome remodelers comprises nine proteins and is characterised by the presence of two chromodomains [3]–[5]. Three CHD proteins (CHD3, CHD4 and CHD5) share a similar domain structure that includes tandem PHD finger and chromo-domains (Fig. 1A). Sequence conservation between these proteins is highest in the catalytic domain (Fig. 1B). A higher sequence variation is evident outside of the catalytic domain (here referred to as the N- and C-terminal regions). In particular, CHD5 possesses a 181 amino acid stretch within the C-terminal region (aa 1556 to 1740) that is unique to CHD5.

The PHD fingers of both CHD4 and CHD5 have been demonstrated to bind the unmodified histone H3 tail *in vitro* [6], [7]. Binding is abrogated by methylation of certain lysine residues such as the di- or tri-methylation of lysine 4 (H3K4me2/3) [6]–[10].

The nucleosome remodeling activities of CHD3 and CHD4 have been extensively analysed *in vitro*. The sequence similarity between the ATPase/helicase domains of CHD3, CHD4 and CHD5 indicates that CHD5 might also be a nucleosome remodeler. However, recent results suggest that sequence similarity of the catalytic domain is a poor predictor of nucleosome remodeling properties as these properties are subject to tight regulation by intramolecular interactions [11]–[13]. Structural analyses of CHD1 and CHD4 have demonstrated that the PHD fingers, the chromodomains and a C-terminal conserved domain of unknown function fold back onto the ATPase domain to modulate its activity [11]–[13]. These findings have revealed that regions outside of the highly conserved ATPase/helicase domains can profoundly influence ATPase and remodeling functions of these enzymes. The nucleosome remodeling activity of CHD5 has so far not been investigated and it remains to be formally demonstrated that CHD5 is an active enzyme.

CHD3 and CHD4 exist in multiprotein complexes referred to as Nucleosome Remodeling and Deacetylation (NuRD) complexes [14], [15]. In neural tissue, CHD5 associates with several NuRD components suggesting that it exists in complexes similar to NuRD [16], [17].

Two CHD proteins have been implicated in cancer: CHD4 has recently been found to be mutated in 17% of serous endometrial tumours [18]. Interestingly, many missense mutations identified in this tumour map to the catalytic domain of the enzyme suggesting that loss of remodeling activity may contribute to malignant transformation. CHD5 resides within a region commonly deleted in neuroblastoma (1p36). Several studies have suggested that CHD5 is a relevant tumour suppressor inactivated in neuroblastoma [7], [19]–[23]. Indeed, in neuroblastoma loss of heterozygosity often occurs by promoter methylation of the remaining *CHD5* gene. Introduction of recombinant CHD5 into these cells inhibits proliferation, clonogenicity and tumour formation in xenograft assays.

In contrast to CHD3 and CHD4 which are expressed in many cell types, CHD5 has a highly restricted expression pattern. Initially, only neuronal tissues were reported to have robust CHD5 expression [17],[24],[25]. Very recently, CHD5 was shown to be also expressed in mouse testis [26]. CHD5-deficient mice produce sperm with aberrant morphology and insufficient chromatin condensation [26]. The molecular mechanisms of CHD5 function during spermatogenesis are not known. It is striking that loss of CHD5 function in differentiating germ cells is not rescued by CHD3 and CHD4 which are expressed at high levels in testis tissue [26].

Spermatogenesis is a strictly regulated process that is initiated immediately after birth and involves several mitotic divisions followed by a long meiotic prophase (two weeks in mice and men). Two meiotic divisions then generate haploid round spermatids. Subsequently, spermiogenesis results in the compaction of the sperm head and differentiation of acrosome and sperm tail. During meiotic prophase, chromosomes pair and recombine in order to ensure correct segregation of homologous chromosomes during the first meiotic division. The X and Y chromosomes pair only in short homologous pseudoautosomal regions and form a transcriptionally inactive chromatin domain (XY body) [27]. During postmeiotic development, X and Y remain partially silenced [28], [29]. As spermatid nuclei start to elongate and condense, histones are modified and subsequently replaced by transition proteins and protamines, yielding a compact and completely transcriptionally silenced genome [30], [31]. Finally, morphologically mature sperm are released into the fluid-filled lumen of the seminiferous tubules. In CHD5 deficient mice histone acetylation during spermiogenesis is impaired [26]. This suggests a potential role of CHD5 in the histone-to-protamine switch.

In this study we use recombinant CHD5 protein to demonstrate for the first time that CHD5 possesses nucleosome-stimulated ATPase activity *in vitro*. We find that CHD3/4 and CHD5 have strikingly different expression patterns in mouse brain and testis: CHD3/4 are widely expressed in neurons of different brain areas. By contrast, CHD5 expression is restricted to distinct subpopulations of neurons with defined topography in the cerebellum and the hippocampus. In adult testis, CHD3/4 and CHD5 are expressed in a mutually exclusive manner. CHD3/4 expression is highest in somatic cells and germ cells at early stages of spermato genesis. These cell types are devoid of CHD5 signal. Instead, strong CHD5 expression is detected in cells that have progressed to the later stages of spermatogenesis. Intriguingly, both CHD3/4 and CHD5 associate with pericentromeric regions of both autosomes and sex chromosomes at particular stages of spermatogenesis and colocalise with macroH2A1.2, a histone variant implicated in organising heterochromatin. In addition, both remodelers show highly specific interactions with specific regions on the sex chromosomes. Our data demonstrate that the highly related ATP-dependent nucleosome remodelers CHD3/4 and CHD5 are differentially expressed during spermatogenesis. Their reciprocal expression and association with chromosomes during spermatogenesis indicates that CHD3/4 remodelers are replaced by CHD5 to exert a specialised function in the context of spermatid chromatin.

## Results

### CHD5 has nucleosome-stimulated ATPase activity

We sought to test if CHD5 has nucleosome remodeling activity *in vitro* by assaying ATPase activity of recombinant CHD5. We generated a baculovirus expressing Flag-tagged CHD5 and used Flag affinity chromatography to purify recombinant protein from extracts of infected cells (Fig. 1C). Proteins were bound to anti-Flag affinity resin, extensively washed and eluted with an excess of Flag peptide. Silver staining of the eluate revealed a prominent band with an apparent molecular mass greater than 170 kDa (Fig. 1C, lane 2). We confirmed the identity of this polypeptide as CHD5 by Western blot analysis (Fig. 1C, lane 1).

We next subjected recombinant CHD5 to an *in vitro* ATPase assay (Fig. 1D). Several CHD family members show robust ATPase activity only in presence of DNA or nucleosomes. Therefore, we included DNA or polynucleosomes in some reactions. CHD5 displayed a dose-dependent basal ATPase activity in the absence of DNA and nucleosomes. Addition of DNA did not stimulate basal ATPase activity. By contrast, addition of polynucleosomes increased CHD5 ATPase activity two- to three-fold. These results suggest that CHD5 has nucleosome-stimulated ATPase activity *in vitro*.

### Expression of CHD5 isoforms in mouse brain and testis

**Figure 2:**
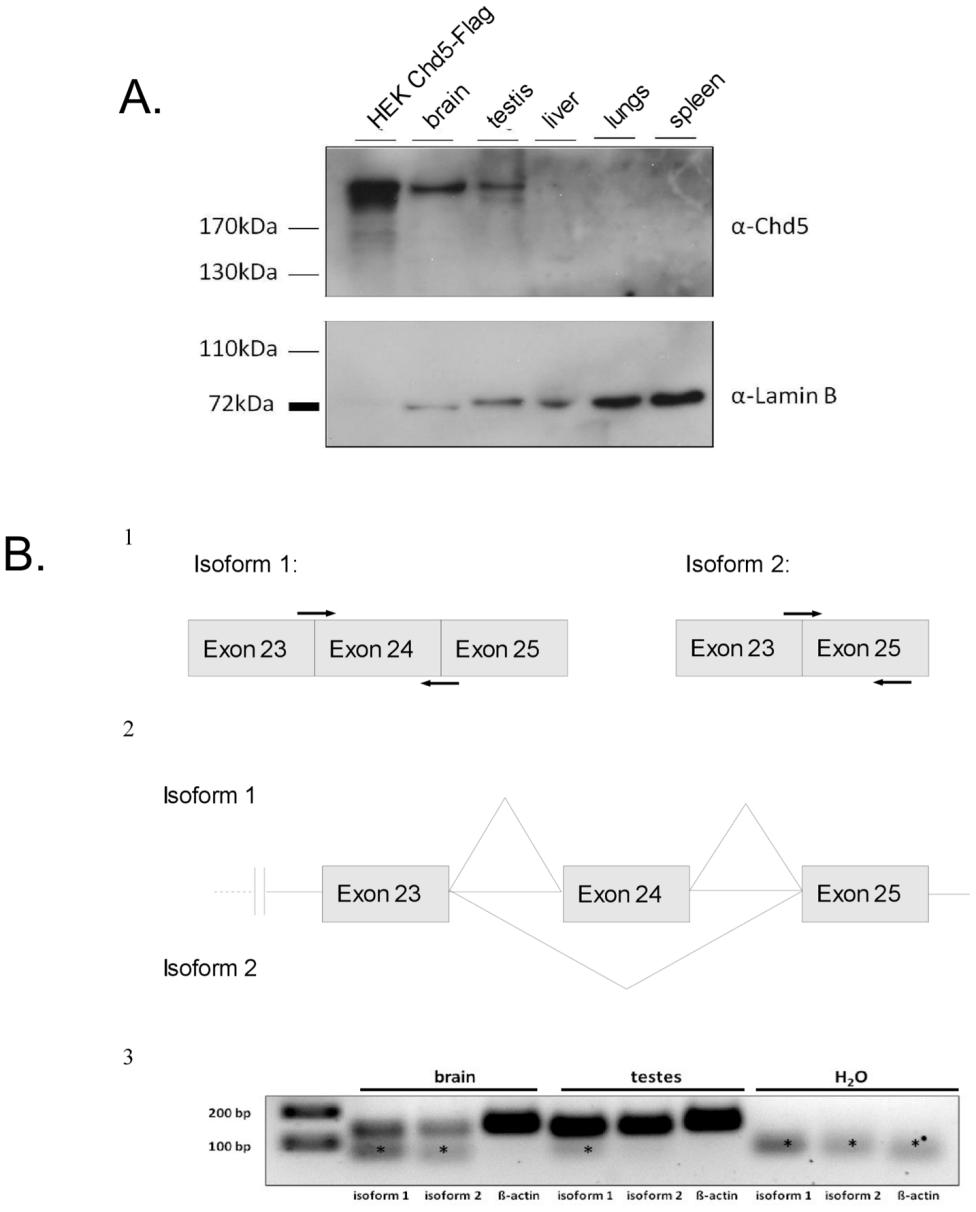
CHD5 protein is expressed in brain and testis. (A) CHD5 protein is expressed in mouse brain and testis. Western blot was performed with CHD5 antibody on lysates from different mouse tissues. Extracts from HEK cells transiently transfected with CHD5-Flag served as a positive control. Lamin B was used as a loading control. (B) Two different CHD5 isoforms are expressed in mouse brain and testis. Panel 1: schematic representation of the location of the PCR primers used to detect CHD5 cDNA of isoforms 1 and 2. Panel 2: schematic representation of part of the CHD5 gene structure showing the two different putative splicing variants. Exons 23, 24 and 25 are indicated. Horizontal lines are introns. Diagonal lines indicate splicing. Panel 3: RT-PCR detecting expression of different CHD5 isoforms in mouse testis and brain. H_2_O: negative control. Asterisk: primer dimers.

In agreement with recently published data, we readily detected CHD5 protein expression in extract derived from mouse brain and testis whereas CHD5 was not detected in liver, lung or spleen extracts (Fig. 2A) [9], [17], [18]. To extend this result we compared *Chd5* mRNA levels in mouse brain and testis. In the mouse, two isoforms of *Chd5* mRNA are predicted which differ in the inclusion of exon 24. We considered that the expression of CHD5 protein in mouse testis might be due to the tissue-specific expression of one of these two isoforms. We made use of two sets of PCR primers to distinguish between the two isoforms. RT-qPCR analysis demonstrated that both mRNA isoforms exist and are expressed in both tissues (Fig. 2B). We have detected no evidence for tissue-specific expression of either isoform. Taken together, these results suggest that two CHD5 isoforms are expressed in brain and testis.

### CHD3/4 and CHD5 expression in brain

**Figure 3:**
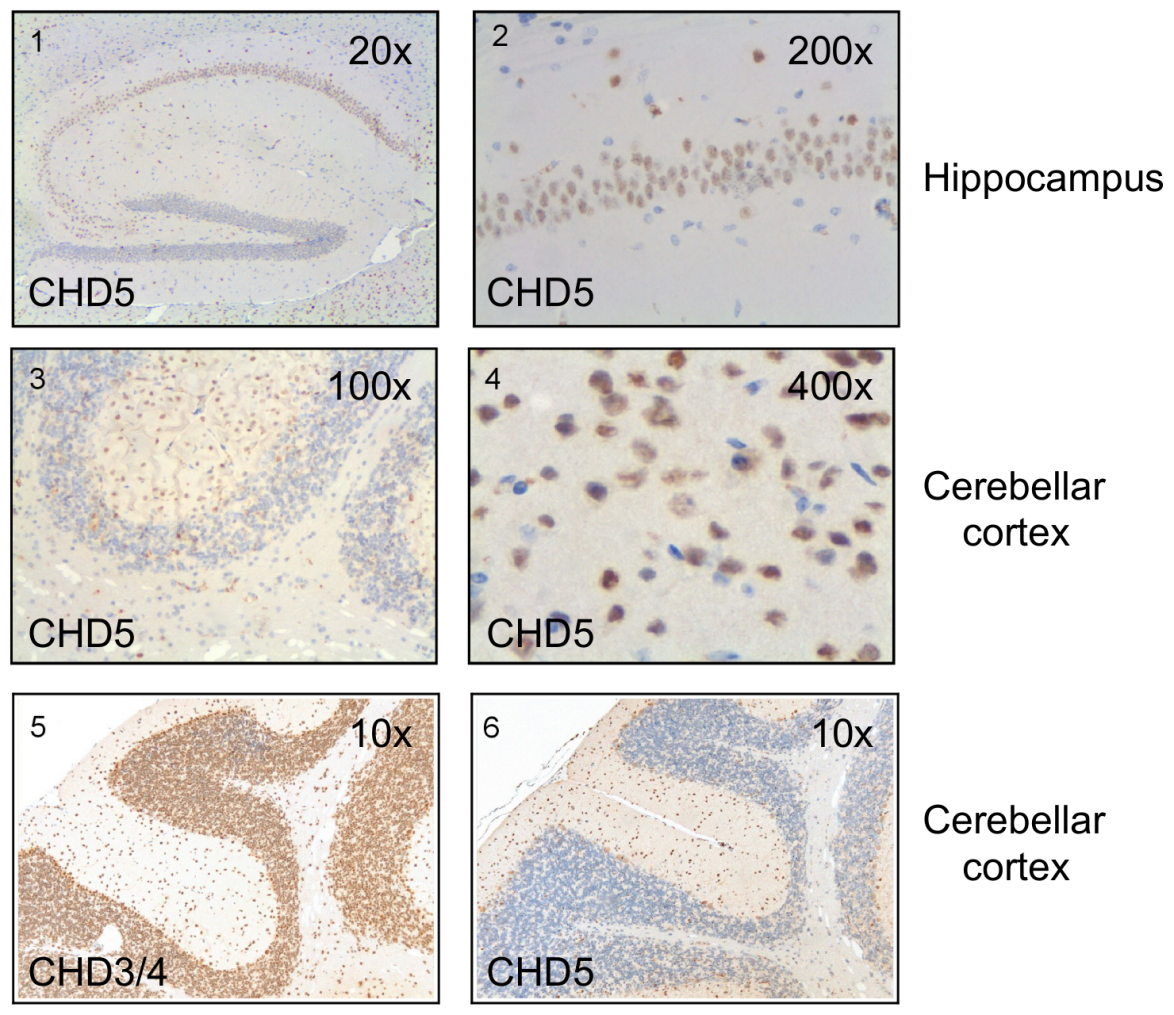
CHD5 protein is expressed in a specific subset of neurons in the brain whereas CHD3/4 is widely expressed. Mouse brain sections were immunostained with antibodies against CHD5 or CHD3/4 and antibody binding was detected using a peroxidase labeled secondary antibody and 3,3'-diaminobenzidine (DAB) as a substrate. (1) CHD5 positive neurons in the hippocampus (20× magnification). (2) 200× magnification of CHD5 positive hippocampal neurons. (3) CHD5 positive neurons in the cortex of the cerebellum (100× magnification). (4) 400× magnification of CHD5 positive cerebellar neurons. (5) and (6) consecutive sections of cerebellum showing CHD3/4 (5) and CHD5 (6) expression.

Brain and testis are composed of a multitude of different cell types. In order to identify CHD5 positive cell types in both organs and to compare the expression pattern of CHD5 with that of the closely related CHD3 and CHD4 proteins we performed immuno histochemistry using specific polyclonal antibodies on paraffin sections of formalin-fixed mouse tissues. We used a CHD5 antibody that binds an epitope that is specific to CHD5. By contrast, the CHD4 antibody recognises an epitope that is shared between CHD3 and CHD4. Accordingly, cells and structures stained with this antibody will be referred to as CHD3/4 positive. In agreement with previous studies, CHD5 signal was detected in the nuclei of neurons of the mouse hippocampus with a restricted expression pattern predominantly in the neuron band of regions CA1 to CA3 (Fig. 3, panels 1 and 2). CHD5 expression was not detected in glial cells. In addition, we detected CHD5 in the nuclei of a restricted subset of neurons in the cerebellar cortex localized in the molecular layer (Fig. 3, panels 3 and 4). Also in other areas of the mouse brain, CHD5 was found in subpopulations of neurons (not shown). To directly compare the expression of CHD3/4 and CHD5 in the cerebellar cortex we stained consecutive sections with CHD3/4 (Fig. 3, panel 5) and CHD5 antibody (Fig. 3, panel 6), respectively. In contrast to CHD5, CHD3/4 displayed a widespread expression in the nuclei of neuronal cells of the brain, including all regions and layers of the hippocampus and the cerebellum (Fig. 3, panels 5 and 6, and data not shown).

### CHD3/4 and CHD5 expression in testis

**Figure 4:**
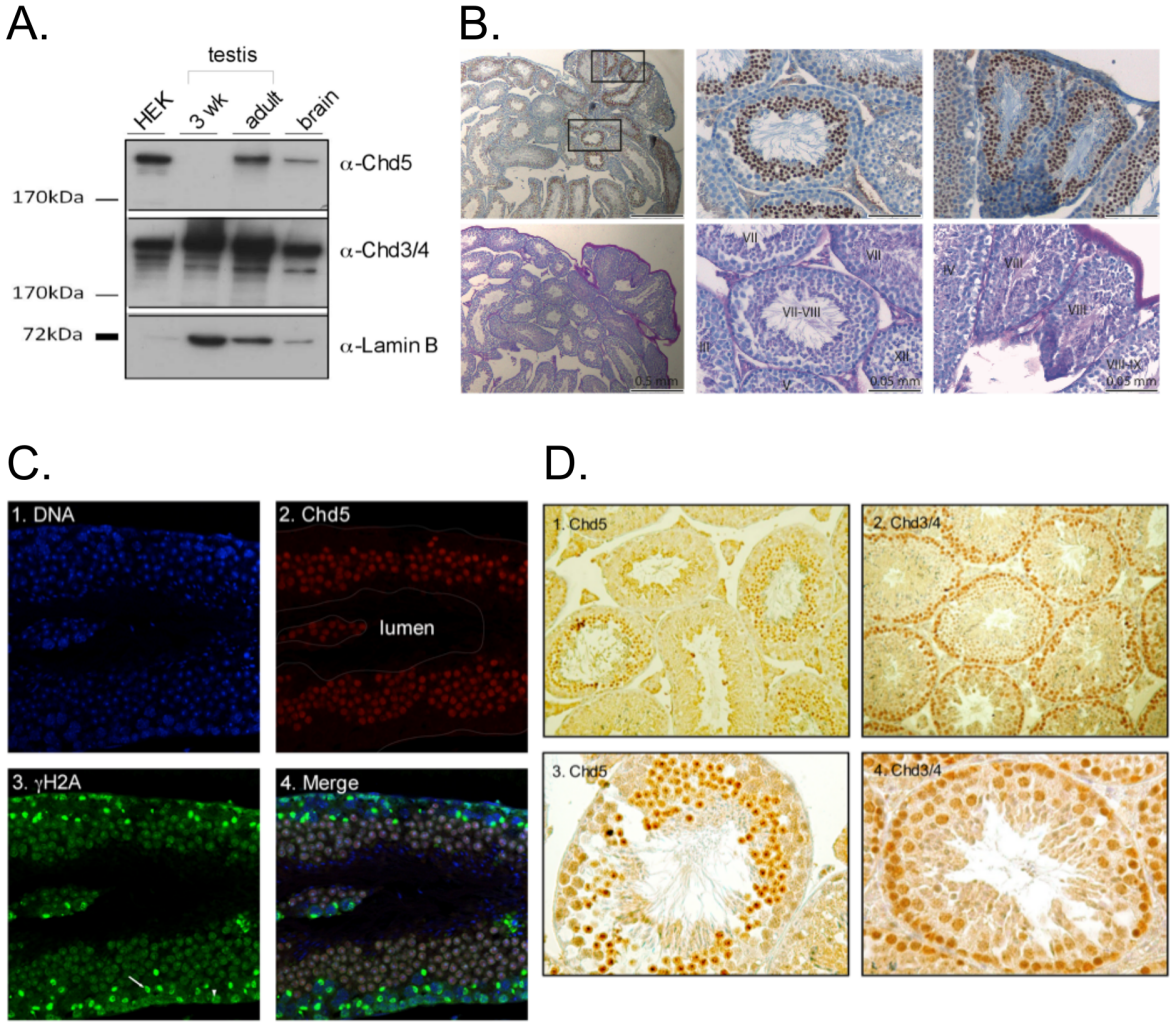
Expression of CHD3/4 and CHD5 is reciprocally regulated during spermatogenesis. (A) Western blot analysis CHD3/4, CHD5 and lamin expression in lysates from 3 week-old and from adult mice. Extracts from HEK cells expressing recombinant CHD5-Flag and extracts from murine brain were used as positive controls for CHD5 expression. Antibodies used for Western blot are indicated. (B) CHD5 protein is maximally expressed in round spermatids at Stages VII–VIII of the spermatognenic cycle. Upper images show results of the immunostaining and the bottom images show the PAS staining pattern of a section directly adjacent to the section shown above it. Left panel: low magnification (scale bars indicate 0.5 mm) of a mouse testis containing seminiferous tubules in different spermatogenic stages. Middle and right: higher magnification of the areas (scale bars indicate 0.05 mm) marked in the left panel, roman numerals indicate the stages of the spermatogenic cycle. (C) CHD5 is expressed in postmeiotic cells. Immunofluorescence analysis was performed using CHD5 antibody and visualized with confocal microscopy. Panel 1: Nuclei stained with DAPI. Panel 2: CHD5 staining, the outline of the tubule as seen in panel 1 is indicated with white lines. Panel 3: phosphorylated H2AX (γH2AX) staining. Spermatocytes in late meiotic prophase (pachytene and diplotene), show intense staining in a subregion of the nucleus (XY body) (arrow). Earlier spermatocytes (at the more basal part of the tubule) show nucleus wide γH2AX staining (arrowhead). Panel 4: overlay of the DAPI, CHD5 and γH2AX channels. (D) CHD5 and CHD3/4 expression at different stages of spermatogenesis. Panel 1: CHD5 is expressed in post-meiotic cells (100× magnification). Panel 2: CHD3/4 is expressed in the area where spermatogonia, Sertoli cells and spermatocytes are present (100× magnification). Panel 3: CHD5 immunostaining (250× magnification). Panel 4: CHD3/4 immunostaining (250x). Formalin-fixed paraffin-embedded tissue sections were immunostained with the antibodies indicated.

We compared expression levels of CHD4 and CHD5 in testis from 3 week old and adult mice by Western blot (Fig 4A). Whereas CHD3/4 protein was readily detectable in both extracts, CHD5 expression was restricted to adult testis. This shows that CHD5 expression is upregulated during testis development and suggests that CHD5 might be expressed in post-meiotic cells.

In order to define the cell types expressing CHD3/4 and CHD5 we analysed cross-sections of testis tubules by immunohistochemistry. In mice, spermatogenesis is intitiated in waves, and the cells move from the basal compartment towards the lumen of the seminiferous tubule during their development. The strict timing of the different steps in spermato genesis, in combination with regular entrance of a new group of differentiating spermato gonia into the process, leads to an ordered association of cells. In mice, twelve different cellular associations or stages (indicated by roman numerals) have been defined [32]. This means that in a cross section of a mouse testis, 12 different associations of spermatogenic cells can be observed in the tubules. The different stages can be identified by Periodic acid-Schiff (PAS) staining. Clearly, not all cross sections of seminiferous tubules showed cells positive for CHD5 (Fig. 4B, left panel). In addition, interstitial cells were all negative for CHD5 (Fig. 4B, left panel). Zooming in on CHD5 positive tubules (Fig. 4B, middle and right panels) revealed that CHD5 expression was excluded from nuclei in the basal part of the seminiferous tubules, indicating that the protein is not expressed in Sertoli cells, spermatogonia, and spermatocytes. In Stage III–VIII sections, CHD5 expression was detected in small round nuclei, representing the postmeiotic round spermatids, that locate in the upper third of the seminiferous tubule epithelium. CHD5 expression was highest in the late round spermatids (stages VII and VIII), and disappeared early during spermatid elongation.

To verify that CHD5 expression is restricted to postmeiotic cells, we costained tubule crossections with CHD5 and γH2AX antibodies. γH2AX is a variant of H2AX that is phosphorylated at Ser139. This modification marks the chromatin surrounding DNA double strand breaks, which are formed early during meiotic prophase [33]. In addition, γH2AX is highly enriched on the chromatin of the X and Y chromosome pair during pachytene [33]. This area of the nucleus is transcriptionally silent and known as the sex body, or XY body (for review see [34]). The results clearly show that CHD5 is only expressed in γH2AX negative postmeiotic cells (Fig. 4C).

Comparison of CHD3/4 and CHD5 in mouse testis revealed an unexpectedly complementary expression pattern (Fig. 4D). Sertoli cells, spermatogonia and spermatocytes, which showed no detectable CHD5 expression, displayed the strongest CHD3/4 signals. CHD3/4 expression was very low or undetectable in round spermatids and in later stages of spermatogenesis. Interestingly, as described above, CHD5 showed the exact opposite expression profile, with its expression starting in early round spermatids. Both, CHD3/4 and CHD5 were not detectable in condensed spermatids, nor in mature sperm released into the lumen.

### Chromosomal associations of CHD3/4 during meiotic prophase

**Figure 5:**
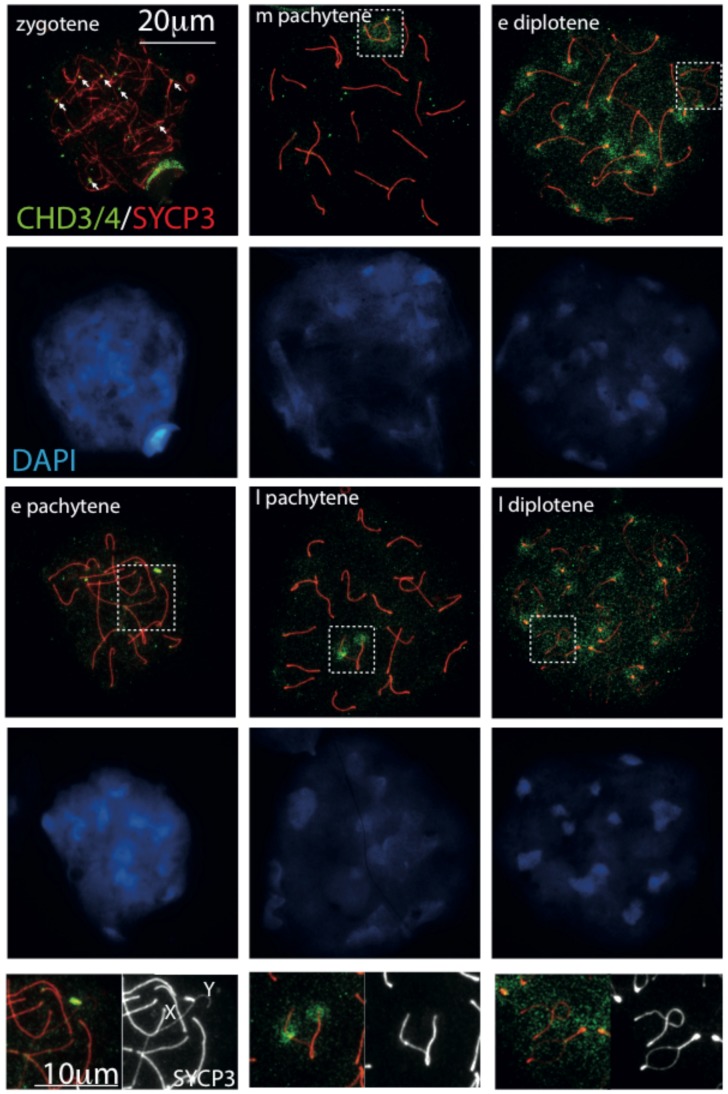
Localization patterns of CHD3/4 in spread nuclei of spermatocytes. Spread wild type mouse spermatocyte nuclei immunostained for CHD3/4 (green), SYCP3 (red), and counterstained with DAPI (blue). In wild type spermatocytes, CHD3/4 is most enriched on some ends of axial elements in zygotene (white arrows), then mainly on the pseudoautosomal region (PAR, P) in early (e) pachytene (see also enlargements at the bottom). Subsequently, all XY chromatin is somewhat enriched in mid (m) pachytene, followed by more specific restriction to the par and the X centromere in late (l) pachytene (enlargement on the bottom of the middle panel). In early and late diplotene, CHD4 enrichment is observed on centromeric heterochromatin, and more evenly distributed on the XY body (enlargement on the bottom of the right panel). XY bodies are indicated with dashed rectangles in the images. In the enlargements, X and Y indicate the axes corresponding to the X and Y chromosome, respectively.

To study the intranuclear localization patterns of CHD3/4 and CHD5 in spermatogenic cell types in more detail, we performed immunocytochemistry on spread nuclei from adult mouse testes. In spermatocytes, progression of meiotic prophase can be assessed by making use of an antibody that recognizes a component (SYCP3) of the protein complex (synaptonemal complex) that physically connects the chromosome axes as they pair, thereby achieving synapsis. When all chromosomes have completed synapsis, the pachytene substage has been reached, and 19 autosomal synaptonemal complexes can be observed as thick lines (Fig. 5). The X and Y chromosomal axes synapse only in the short pseudoautosomal regions (PAR), and the rest of the chromosomal arms remain unsynapsed and displays a thinner SYCP3 line. The X and Y chromosome form the socalled XY body, which can also be recognized as a somewhat more DAPI-dense region in the nuclear spreads. Interestingly, while CHD5 is not detected in spermatocytes (see above and data not shown), CHD3/4 shows a dynamic pattern of localization during spermatocyte development. At leptotene and zygotene, 150–250 meiotic DSBs are induced by the transesterase SPO11. This enzyme generates approximately 200–250 DSBs and repair of these breaks is instrumental for correct chromosome pairing [35], [36]. In these early meiotic prophase cells, CHD3/4 levels were low and dispersed over the chromatin, with clear foci (4 to 8) on the ends of a few chromosomal axes (Fig. 5). In early pachytene, CHD3/4 was highly enriched along the short synapsed stretch of the XY pair, corresponding to the pseudoautosomal region. Somewhat later during pachytene, the rest of the XY body also showed enrichment, in particular the (peri)centromeric region of the X. In diplotene, CHD3/4 was no longer clearly enriched on the XY body, but accumulated more on the DAPI-dense (peri)centromeric chromatin of the autosomes (Fig. 5).

**Figure 6:**
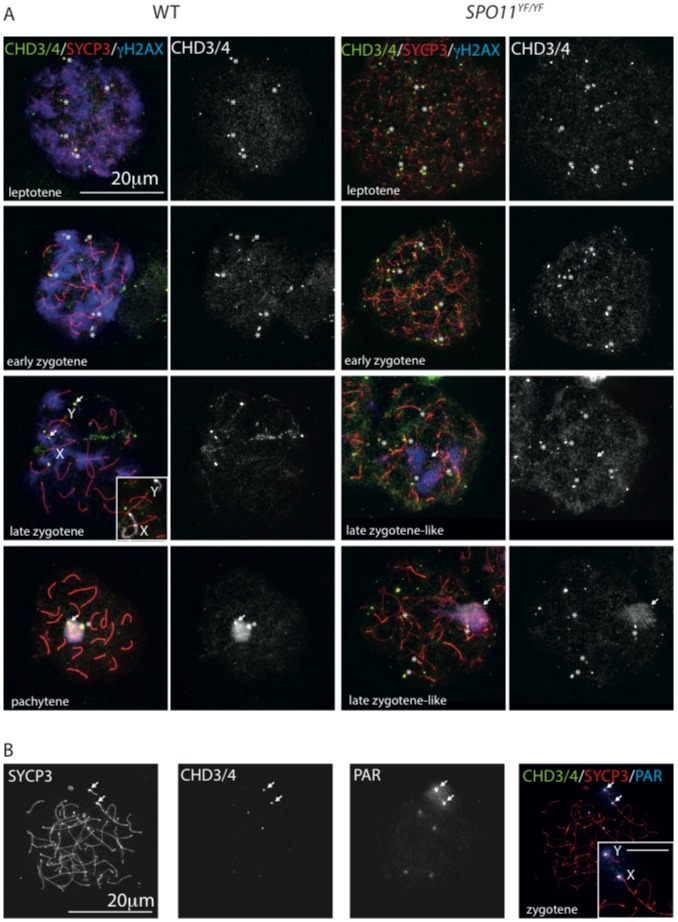
CHD3/4 localisation in spermatocytes is independent of SPO11. (A) Spread wild type (left) and *Spo11* null (*Spo11^YF/YF^*, right) spermatocytes stained for CHD3/4 (green), SYCP3 (red), and γH2AX (blue), the single CHD3/4 staining is shown in white to the right of each merge. In wild type nuclei, CHD3/4 does not colocalise with γH2AX in leptotene and zygotene. Specific foci accumulate on some chromosome ends (asterisks indicate examples), in particular on the pseudoautosomal region (PAR) of both X and Y, as can be observed in the late zygotene nucleus (white arrows). The inserted image shows part of the same nucleus with the X and Y chromosomal axes in white, for easy recognition. In pachytene, γH2AX and CHD3/4 colocalize on the XY body whereby CHD4 is particularly enriched on the PAR (white arrow) and the X centromere (asterisk). In *Spo11* null spermatocytes enrichment of CHD3/4 is observed on some chromosome ends in early zygotene (asterisks indicate examples), and in the pseudo XY body (white arrow) of later zygotene-like spermatocytes. (B) Spread *Spo11* null zygotene spermatocyte stained for SYCP3 and CHD3/4, and hybridized with a BAC probe recognizing the PAR. In the merge, an enlargement of the region encompassing the X and Y is shown (scale bar indicates 5 µm). In this nucleus CHD3/4 stain the PAR of the X and Y, but also mark the ends of 4 additional chromosome ends, that are also recognized, to a lesser extent, by the PAR probe. It is not clear what causes this crossreaction (see Materials and Methods).

CHD3/4 is involved in regulating DNA double-strand break (DSB) repair [37]. In addition, the protein accumulates at sites of DNA damage [37]–[39]. The diffuse CHD3/4 staining pattern in leptotene and zygotene, with only a few foci at chromosome ends does not indicate that the protein is directly recruited to the >100 sites of meiotic DSB repair on the chromosomal axes [36], [40]. Nevertheless, we wished to asses whether the accumulation of CHD3/4 on chromatin in meiotic prophase cells could be a response to the presence of meiotic DSBs. Meiotic DSB formation is accompanied by nucleus wide formation of γH2AX [33]. As DSB repair and chromosome pairing progress, γH2AX gradually disappears from autosomes, but accumulates on the XY body [33]. In the absence of the enzyme (SPO11) that generates the meiotic DSBs, chromosome pairing is severely disrupted and γH2AX accumulates only in a small transcriptionally silenced subnuclear region named pseudo XY body [41], [42], most likely triggered by the combination of the presence of a few spontaneous DSBs and asynapsed chromatin [43]. This region only rarely encompasses the X and Y chromosomes, but covers part of the asynapsed chromatin [44], [45]. In *Spo11* null mutant mice (*Spo11^YF/YF^*), spermatocytes arrest at a stage corresponding to early pachytene in wild type, but with a zygotene-like appearance, similar to what has been observed in *Spo11* knockout mice [41]–[43]. We performed triple stainings for γH2AX, SYCP3 and CHD3/4 in wild type and *Spo11* null mutant spermatocytes and observed that there was no clear colocalisation between CHD3/4 foci and the global accumulation of γH2AX in wild type leptotene and zygotene nuclei (Fig. 6A, left panel). CHD3/4 only colocalises with γH2AX in the XY body of early pachytene wild type spermatocytes (Fig. 6A, left panel). In *Spo11* null mutant leptotene and zygotene nuclei, we still observed CHD3/4 foci at the ends of some of the axes (Fig. 6A, right panel), and in the majority of the later stage nuclei we also observed CHD3/4 enrichment on the chromatin area that encompassed the pseudo XY body, as evidenced by enrichment for γH2AX (Fig. 6A, right panel). Since the pseudo XY body only rarely includes the X or Y chromosome, we determined whether any of the foci observed on ends of axial elements actually represented the pseudoautosomal regions, by combining SYCP3 and CHD3/4 immunostaining with a FISH for the PAR region. Figure 6B shows a representative result in which it is clear that CHD3/4 is enriched at the PAR in zygotene *Spo11* null spermatocytes, and at some other chromosome ends that react weakly with the PAR probe for unknown reasons. Also in wild type nuclei, at a very late zygotene stage, the PAR of both the X and the Y chromosome accumulates CHD3/4, before the two chromosomes synapse (Fig. 6A, left panel).

### Chromosomal associations of CHD5 during spermatid differentiation

**Figure 7:**
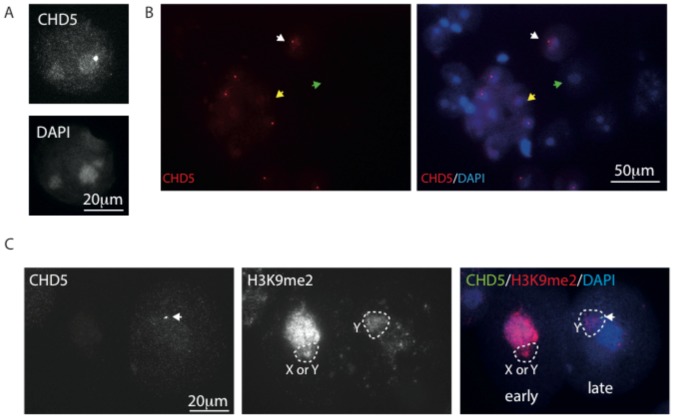
CHD5 is enriched in pericentromeric chromatin of round spermatids. (A, B) Spread nuclei of spermatids immunostained for CHD5 and counterstained with DAPI. An example of a round spermatid nucleus that is positive for CHD5 expression and displays the high intensity focus in the chromocenter is shown in A and such an example is also indicated with a white arrow in B, the yellow arrow indicates a spermatid nucleus that expresses CHD5, but does not have the extra CHD5 focus. The green arrow indicates a CHD5-negative round spermatid nucleus. (C) Spread nuclei of spermatids immunostained for CHD5 and H3K9me2, and counterstained with DAPI. The nuclear area encompassing the chromatin of the sex chromosome is indicated with a dashed line H3K9me2 marks the chromocenter and the sex chromosome in the left (early) round spermatid nucleus, and mainly the sex chromosome in the right (late) round spermatid nucleus. CHD5 level is very low in the left nucleus, but clearly enriched in the chromocenter and the focus (indicated with a white arrow) adjacent to the chromocenter and Y chromosome chromatin in the right nucleus.

In round spermatids, CHD3/4 expression was absent (see above and data not shown), and CHD5 signal could be detected in some of the spermatid nuclei. CHD5 was enriched in the round DAPI-dense area named chromocenter, that contains the (peri)centromeric chromatin (Fig. 7A, B). In some of the round spermatids we observed a spot within or at the edge of the chromocenter that was most intensely stained (Fig. 7A, B). The spot is also adjacent to the area that encompasses the sex chromosome, which can be recognized by a distinct DAPI intensity, that is somewhat more intense than the noncentromeric chromatin, and less intense than the chromocenter. The sex chromosome in spermatids is known to carry enrichments for some specific histone modifications, such as for example H3K9me2, which can be observed in a subfraction of the spermatids as indicated in Figure 7C. In spermatids, γH2AX foci mark DNA breaks that are transiently present in elongating spermatids. At this stage, CHD5 expression is no longer detectable (not shown), indicating that also CHD5 accumulation in round spermatids is not induced by the presence of DNA damage.

**Figure 8:**
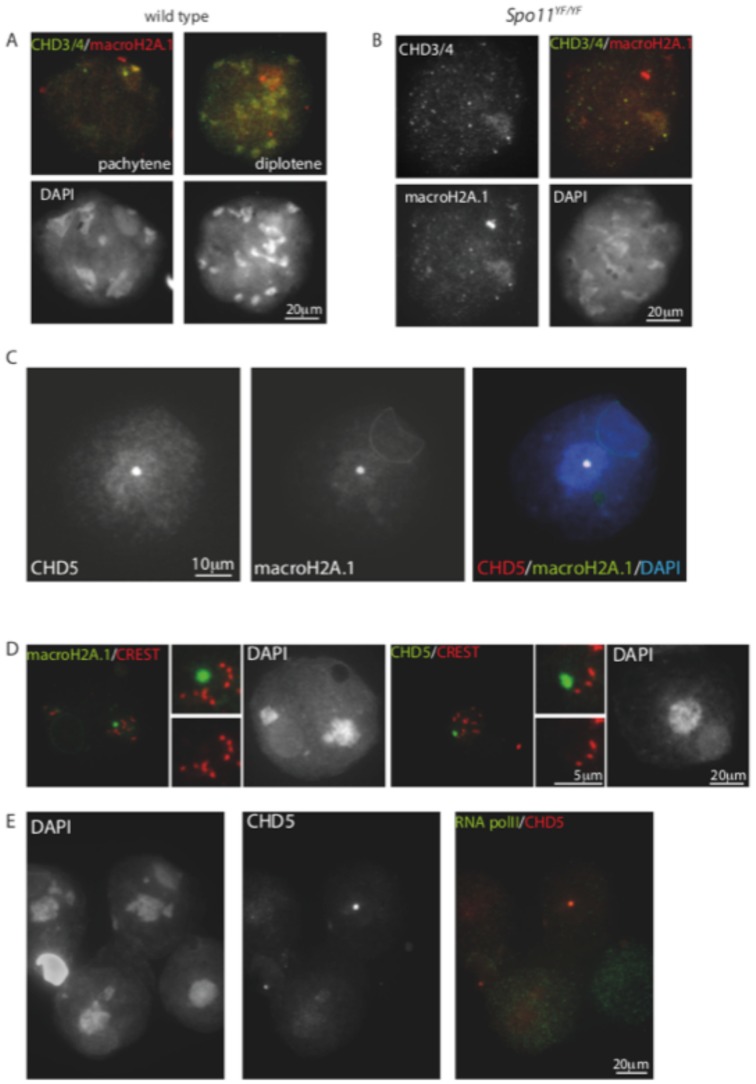
macroH2A colocalizes with CHD3/4 in spermatocytes and with CHD5 in spermatids. (A,B) Spread wild type (A) and *Spo11* null (*Spo11^YF/YF^*) (B) spermatocyte nuclei stained for MacroH2A.1 and CHD3/4. Colocalisation on the PAR and the X centromere is observed in pachytene, but not in diplotene nuclei of wild type spermatocytes. In the absence of functional SPO11, some zygotene-like nuclei show enrichment of both CHD4 and macroH2A.1 in the pseudo XY body. (C) Spread spermatid nucleus stained for CHD5, and MacroH2A, colocalisation of the CHD5/MacroH2A focus in the chromocenter is clear in the merge with DAPI (right image). (D) Spread spermatid nuclei stained with macroH2A (green) and CREST (red, left panel) or with CHD5 and CREST (right panel). The enlargements in the middle of each panel clearly show that the macroH2A.1/CHD5 spot in the chromocenter does not colocalise with CREST marking of the centromeres. (E) Spread spermatid nuclei costained for CHD5 and RNA polymerase II. Both high and low levels of RNA polymerase II can be observed in nuclei that express CHD5.

The enrichment of CHD3/4 in the XY body and on centromeric heterochromatin in spermatocytes, and the localization of CHD5 on the chromocenter and an extra spot in round spermatids is very reminiscent of the staining pattern that has been described for macroH2A1.2 [46]. This study has shown that the focus of macroH2A1.2 enrichment in round spermatids colocalises with the (peri)centromeric region of the Y chromosome. Based on this information, we compared the expression of macroH2A1.2 to that of CHD3/4 and CHD5 in spermatocytes and spermatids, respectively. In wild type pachytene nuclei, we indeed observed that CHD3/4 is enriched in areas that are also enriched for macroH2A1.2 (Fig. 8A). However, in diplotene, when most CHD3/4 was lost from the XY body, macroH2A.1 was still enriched on the PAR and the centromeric regions. In *Spo11* null spermatocytes, we observed colocalisation of macroH2A.1 and CHD3/4 in the pseudoXY body, whereby macroH2A.1 was usually more clearly enriched than CHD3/4 (Fig. 8B).

In accordance with previous observations [46], we observed macroH2A.1 in a focal spot at the border of the chromocenter in approximately half of the round spermatids that were scored (115/217). Colocalisation with CHD5 was observed in 54 of the 115 spermatids that were macroH2A.1-positive (Fig. 8C). It has been shown that the macroH2A.1 spot in the chromocenter is observed specifically in spermatids that contain the Y chromosome [46], and it was suggested that the spot corresponds to the Y centromere. To verify whether both CHD5 and macroH2A.1 colocalise on the Y centromere, we co-stained round spermatids for the centromere marker CREST and either CHD5 or macroH2A.1. For both proteins we observed no specific colocalisation with CREST (Fig. 8D), indicating that the proteins are enriched on a noncentromeric region of the Y chromosome.

Since CHD5 is not enriched on all round spermatids, we wondered if the level of CHD5 could be related to the overall level of gene transcription. We used an antibody against RNA polymerase II as a marker for overall transcriptional activity in combination with CHD5, but observed CHD5 accumulation in the chromocenter and the Y-chromosome specific spot both in nuclei with high and with low levels of RNA polymerase II (Fig. 8E)

## Discussion

### CHD5 is a nucleosome stimulated ATPase

CHD3 and CHD4 can remodel nucleosomes in an ATP-dependent manner *in vitro*. Recently, regions flanking the ATPase domain of CHD proteins have been demonstrated to interact with the ATPase domain and to regulate enzyme activity by modulating the interaction of the enzyme with DNA and nucleosome substrates [11]–[13]. The ATPase domains of CHD3, CHD4 and CHD5 are highly conserved. However, amino acid sequence conservation in the flanking regions is not as high. In particular, CHD5 contains a unique 181 amino acid stretch in the C-terminus suggesting possible differences in intramolecular interactions and ATPase regulation. Here, we have shown that recombinant CHD5 hydrolyses ATP *in vitro*, thus demonstrating for the first time that CHD5 is indeed an active ATPase enzyme. Previous work has shown that recombinant CHD4 ATPase is stimulated to a similar extent by both free DNA and nucleosomes, a property that is shared by ATP-dependent nucleosome remodelers of the SWI/SNF family [47]. In contrast, we find that CHD5 ATPase activity is stimulated by nucleosomes but not significantly by free DNA. This suggests differences between CHD4 and CHD5 in the way ATPase activity is regulated in the presence of different DNA substrates. Since addition of nucleosomes significantly activates the CHD5 ATPase, it is likely that CHD5 remodels nucleosomes *in vitro*.

Why do vertebrates express three remodeling enzymes (CHD3, CHD4 and CHD5) that are highly related in their amino acid sequences? Moreover, all three enzymes reside in multisubunit protein complexes with similar subunit composition [16], [17]. One possibility is that these enzymes perform related but different chromatin remodeling reactions. The differences in ATPase activities described above are consistent with such a hypothesis. Related enzymes often show distinct expression patterns and provide tissue-specific functions. Indeed, the expression patterns of CHD3/4 and CHD5 are markedly different. Initially, CHD5 was found to be exclusively expressed in tissues of neural origin [17], [24], [25]. This data is supported by comparative analysis of CHD3, CHD4 and CHD5 mRNA expression (Figure S1). Whereas CHD3 and CHD4 are expressed at high levels in 17 mouse tissues examined, CHD5 showed significant expression in brain only. This analysis did not detect significant CHD5 mRNA expression in testis. In contrast, our semi-quantitative measurement of CHD5 mRNA in testis extract suggests that CHD5 expression in testis is even stronger than in brain. The reasons for this discrepancy are currently unclear. However, Zhuang and colleagues [26] have recently used quantitative RT-qPCR to compare CHD5 expression levels in brain and testis. They have found that CHD5 mRNA is four times more abundant in testis extract. This finding agrees well with our results. In addition, the analysis of CHD5 protein expression in testis by Western blot, immunohistochemistry and immunofluorescence confirms robust CHD5 expression in this tissue (our study and [26]).

In this study, we have also demonstrated the existence of two alternatively spliced CHD5 mRNA isoforms that had been predicted by sequence analysis. Both isoforms are expressed in brain and testis. They differ by the inclusion/exclusion of exon 24. The predicted difference in the molecular masses of the resulting proteins is too small to be separated by SDS-PAGE and Western blot analyses. Therefore, it is currently unknown to what extent both isoforms are expressed at the protein level.

In the brain, CHD5 is expressed as a nuclear protein in distinct subsets of neurons, with particularly clear topographical patterns in the hippocampus and cerebellum. We did not detect CHD5 in non-neuronal cell types. Our data agree well with previous analyses of CHD5 expression in human brain [17], [24]. We also compared for the first time the expression patterns of CHD4 and CHD5 in the mouse brain. It is evident that CHD3/4 expression is broader than CHD5, encompassing most neuronal cells. In the molecular layer of the cerebellum most neurons show immunostaining for both CHD3/4 and CHD5 as evident from consecutive sections. These data suggest that at least some neural populations coexpress both CHD3/4 and CHD5. Preliminary analyses of human cerebellum suggest expression patterns of CHD3/4 and CHD5 similar to those observed in mouse cerebellum (data not shown).

Our analysis of CHD3/4 and CHD5 expression in mouse testis has revealed that expression of both remodelers is developmentally regulated during spermatogenesis. CHD3/4 is expressed in both germ cells and Sertoli cells, whereas CHD5 was detected in germ cells only, indicating a germ cell specific function of CHD5. Indeed, we found that the patterns of CHD3/4 and CHD5 expression during spermatogenesis are mutually exclusive; CHD3/4 is present in spermatogonia and spermatocytes, but not in the postmeiotic spermatids, whereas CHD5 expression initiates during round spermatid development, with a peak in step VIII spermatids, followed by a dramatic reduction to undetectable levels in step IX spermatids. The opposing expression profiles of CHD3/4 and CHD5 during spermatogenesis suggest that CHD5 replaces CHD3/4 to carry out a specialised function. The differences in the biochemical properties of CHD3/4 and CHD5 described above are consistent with this hypothesis.

Spermatogenesis is characterised by a series of dramatic chromatin remodeling events. In meiotic prophase, DNA double-strand breaks are specifically induced at sites that carry a specific histone modification signature, and the H3.1 containing nucleosomes of the transcriptionally silenced XY pair in the XY body are completely replaced by H3.3 containing nucleosomes [48]. This process is also accompanied by dynamic changes in histone modifications. In addition, meiosis-specific histone variants are incorporated in a global manner at different stages of germ cell development and finally, during postmeiotic spermatid elongation, the vast majority of histones are replaced by transition proteins and subsequently by protamine. These chromatin remodeling processes are unique to spermatogenesis and are likely to require specialised chromatin remodeling machines.

Our expression study suggests that CHD3/4 performs a specific function in the pseudoautosomal region during early meiotic prophase. This region is of special interest in the context of meiotic DSB repair since it contains a super hotspot for SPO11 activity, that ensures that a crossover is formed in this small region in all spermatocytes [49]. We observe CHD3/4 foci at the X and Y PAR well before the two chromosomes have associated, in leptotene spermatocytes, independent of SPO11 activity. It might therefore be suggested that CHD3 or CHD4 is involved in the generation of an open chromatin structure at the PAR hotspot, preparing it for very efficient cutting by the isoform of SPO11 that is specifically involved in this function [50]. It is surprising that CHD3/4 does not form foci at the meiotic DSBs, since CHD4 is recruited to laser-induced DSBs in cultured mammalian somatic cells [37]–[39]. Since the recruitment of CHD4 to DSBs in somatic cells is thought to depend on PARylation and result in recruitment of RNF168 and resultant H2A ubiquitylation, it is of interest to note that global H2A ubiquitylaton does not occur in association with meiotic DSB formation [51]. Instead, this modification, and also the PARylating enzyme PARP-2 accumulate at the XY body. Moreover, in *Parp-2* knockout mice, XY body formation appears to be affected and overall crossover formation was reduced [52]. Finally, PARP protein has been reported to bind to a hotspot consensus site [53]. Taken together, our data support the hypothesis that the CHD3/CHD4 staining pattern in zygotene and early pachytene spermatocytes is related to a CHD4 remodeling function in association with the PAR hotspot, and not a function that is directly related to a response to DSBs. The localisation to (peri)centromeric heterochromatin during later meiotic prophase may reflect a function of CHD4 in regulation of transcriptional repression at these sites [54].

The timing of CHD5 expression during spermatogenesis described in this study and the defects of CHD5-deficient sperm described by the Brodeur lab raise questions about the role of CHD5 in the histone-protamine switch and chromatin condensation [26]. At present, our knowledge of the mechanisms contributing to the histone-protamine exchange is limited and the factors involved are only beginning to be characterised. A major role is played by a global wave of histone hyperacetylation (in the absence of active gene transcription), which may lead to a more open chromatin structure that subsequently may facilitate replacement by transition proteins and protamines. Recently, the testis-specific bromodomain protein BRDT which binds acetylated nucleosomes was demonstrated to regulate histone removal and incorporation of transition proteins [55]. In addition, testis-specific H2B variants such as TH2B have been shown to play important roles in the histone-protamine switch [56]. Accordingly, it has been suggested that the whole process may occur without direct involvement of ATP-dependent chromatin remodelers (reviewed by [31]). The preferred localization of CHD5 to (peri)centromeric heterochromatin may also indicate that it is not directly involved in histone removal, but perhaps in ensuring that the centromeric nucleosomes are retained, since these are known to be transferred to the zygote [57]. Moreover, CHD5 expression appears to peak when global gene transcription shuts down, prior to histone removal [58]. Thus, it might also be suggested that CHD5 is involved in mediating this global transcriptional repression, in particular since CHD5 follows the localization pattern of macroH2A.1, which is also known to be involved in transcriptional repression. Alternatively, CHD5 might actively contribute to global histone acetylation as H4 hyperacetylation levels are decreased in CHD5-deficient spermatids [26]. Future functional analyses of CHD3/4 and CHD5 and identification of their specific binding partners in spermatocytes and spermatids, respectively, will help to further delineate the specific functions of these chromatin remodelers in spermatogenesis.

## Materials and Methods

### Generation of CHD5-Flag constructs

A *CHD5* cDNA construct was kindly provided by G.M. Brodeur [20]. This cDNA clone was used as a template for PCR to generate hChd5-Flag/pcDNA3.1/V5-His-TOPO and Chd5-Flag/pVL1392 constructs. hChd5-Flag/pcDNA 3.1/V5-His-TOPO was used to produce CHD5 expressing cell extract whereas Chd5-Flag/pVL1392 was used to generate recombinant human CHD5-Flag.

### ATPase assay

Human recombinant CHD5-Flag was produced using the baculovirus system as decribed in [59]. The recombinant protein was purified by Flag-immunoprecipitation and eluted with Flag peptide (Sigma-Aldrich F3290) according to the manufacturer's instructions. The eluted material was loaded on SDS-PAGE followed by silver staining or anti-CHD5 (Santa Cruz sc-68390 (H-185)) Western blotting. The recombinant protein was then used in the ATPase assay as described in [60].

### Mouse tissue and cell line nuclear extracts

Nuclear extracts were prepared from mouse tissues using a protocol adapted from [61]. Tissues were homogenized in lysis buffer (50 mM tris, 5 mM EDTA, 150 mM NaCl) and centrifuged. Pellets were homogenized in homogenization buffer (10 mM Hepes pH 7.9, 10 mM KCl, 1.5 mM MgCl_2_, 0.1 mM EDTA and 0.1 mM EGTA) and incubated for 15 minutes on ice. NP-40 was added to a final concentration of 3% and extracts were vortexed, incubated for 30 minutes on ice and shortly spun down. Finally, nuclear pellets were resuspended in ice-cold nuclear extract buffer (20 mM Hepes pH 7.9, 420 mM NaCl, 1.5 mM MgCl_2_, 1 mM EDTA and 1 mM EGTA). As a positive control for CHD5 expression, extract from CHD5-Flag overexpressing cells was used. Cell line extract was prepared using a standard protocol for cell lysis. For overexpression, the Hek293 cell line which is derived from human embryonic kidney cells was transiently transfected with CHD5-Flag/pcDNA3.1/V5-His-TOPO. Western blotting of extracts was performed according to standard procedures using the primary antibodies anti mouse-CHD5 rabbit polyclonal (Santa Cruz Biotechnology (M-182) sc-68389) and anti Lamin B goat polyclonal (Santa Cruz Biotechnology (M-20) sc-6217).

### Mouse tissue mRNA extraction and RT-PCR

RNA from mouse tissue was extracted using Trizol (Invitrogen) according to the manufacturer's protocol. 2 mg of RNA (with an optical density between 1.8 and 2.2) was reverse transcribed into cDNA by incubation with 0.5 mg oligo(dT) primers (MWG) and 200 U M-MLV reverse transcriptase (Invitrogen) according to the manufacturer's protocol. Quantitative (Q)PCR was performed using Absolute QPCR SYBR Green Mix (Thermo Scientific) and analyzed by the Stratagene Mx3000P real-time detection system (Agilent). At least two independent experiments were performed and all amplifications were done in triplicate. Relative gene expression was determined by calculating 2^-DDCt^ values. Values were obtained by normalization using the housekeeping gene RPS14 and a non-CHD5 expressing calibration sample. For semi-quantitative RT-PCR, standard PCR reactions using CHD5 isoform-specific primers were performed on cDNA. The PCR primers used for detection of CHD5 and housekeeping genes in mouse tissue are:

b-actin forward: 5'-gatctggcaccacaccttct-3'; b-actin reverse: 5'-ggggtgttgaaggtctcaaa-3; CHD5 isoform 1 forward: 5′-cgtggaaggcatgatgt-3′; CHD5 isoform 1 reverse: 5′-ctttgttatcgcctggc-3′; CHD5 isoform 2 forward: 5′-gtggaaggcgataacaaag-3′; CHD5 isoform 2 reverse: 5′-aggtactcgttcatgttct-3′.

### Immunohistochemistry and immunocytochemistry

Stainings of formalin-fixed and paraffin-embedded mouse tissue sections from testis and brain were performed using standard immunohistochemistry protocols. Endogenous peroxidase activity was blocked in 3% H_2_O_2_ (Merck) in methanol. For staining of mouse testis, 8–10 mm thick deparaffinized sections were boiled in citrate buffer (Dako target retrieval solution citrate pH 6). Unspecific binding of primary and secondary antibody was prevented by blocking with 10% normal goat serum (Dako X0907) and 5% bovine serum albumin (BSA, Sigma A7034) in phosphate buffered saline (PBS). As first antibodies polyclonal rabbit anti mouse-CHD5 (Santa Cruz Biotechnology (M-182) sc-68389) or anti Mi2 (Santa Cruz Biotechnology (H-242) sc-11378) were used. As negative controls, slides were incubated in blocking buffer (3% BSA/PBS-0.1%Tween) without primary antibody. As secondary antibody a biotinylated goat anti-rabbit IgG (Dako, E0432) was used. An amplification step was performed using the ABC kit (Vectastain, Elite PK-6100 standard) together with the chromogen substrate 3,3'-diaminobenzidine (Dako liquid DAB substrate chromogen system K3468) for immunohistochemistry. Sections were counterstained with hematoxylin and mounted with mounting medium. To determine the stage of the cycle of the seminiferous epithelium, adjacent sections were stained with Periodic acid Schiff and analysed according to [32].

For immunohistochemistry on mouse brain, the following modifications were employed: heat-induced antigen retrieval was performed by treating the sections with Trilogy Pretreatment Solution (Cell Marque) for 30 min in a household steamer. For detection of CHD5, polyclonal rabbit anti human-CHD5 (Santa Cruz Biotechnology (H-185) sc-68390) which is also reactive with mouse CHD5 was used. The antibody incubation and washing steps, including prior blocking of endogenous peroxidase activity, were performed using an automated immunohistochemistry apparatus (Autostainer plus; Dako). Detection of bound primary antibodies was via Dako REAL Detection System Peroxidase/DAB+, Rabbit/Mouse (Dako).

For immunofluorescent stainings of mouse testes cryosections, no endogenous peroxidase inhibition step was performed. Primary antibodies were anti mouse-CHD5 rabbit polyclonal (Santa Cruz Biotechnology (M-182) sc-68389) and anti-gH2AX mouse monoclonal (Upstate #05-636, clone JBW301). Slides were incubated with a fluorescent second antibody (Alexa Fluor 546 Goat Anti-Rabbit or 488 Goat Anti-Mouse IgG (H+L), Life Technologies). Sections were embedded with Prolong Gold antifade reagent containing DAPI (Life Technologies P-36931).

Spread nuclei of spermatocytes from wild type and *Spo11^YF/YF^* mutant mice were prepared as described (Peters et al., 1997) and stored at −80C. Thawed slides were washed in PBS (3×10 min), and non-specific sites were blocked with 0.5% w/v BSA and 0.5% w/v milk powder in PBS. Primary antibodies were diluted in 10% w/v BSA in PBS, and incubations were done overnight at room temperature in a humid chamber. Subsequently, slides were washed (3×10 min) in PBS, blocked in 10% v/v normal goat serum (Sigma) in blocking buffer (supernatant of 5% w/v milk powder in PBS centrifuged at 14,000 rpm for 10 min), and incubated with secondary antibodies in 10% normal goat serum in blocking buffer at room temperature for 2 hours. Finally, slides were washed (3×10 min) in PBS (in the dark) and embedded in Prolong Gold with or without DAPI (Invitrogen). First antibodies: rabbit polyclonal anti-SYCP3 (gift from C. Heyting), rabbit polyclonal anti-CHD3/4 and antiCHD5 as described above, rabbit polyclonal anti-macroH2A.1 (Upstate), mouse monoclonal anti-H3K9me2 (Abcam), mouse monoclonal anti-gH2AX (Upstate), RNA polymerase II (Abcam), and human autoantibodies against the centromere (CREST, Cortex Biochem). For secondary antibodies, we used a goat anti-rabbit alexa 488/546 IgG, goat anti-mouse 488 alexa IgG and goat anti-human alexa 555 IgG, respectively. When double-immunostainings were performed with two rabbit polyclonal antibodies, these were performed in consecutive order as follows: CHD3/4 or macroH2A.1+ SYCP3; first SYCP3 (first and second antibody), then CHD3/4 or macroH2A.1, respectively, CHD3/4 or CHD5 + macroH2A.1; first CHD3/4 or CHD5 (first and second antibody) followed by macroH2A.1 Fluorescent images were observed by using a fluorescence microscope (Axioplan 2; Carl Zeiss) equipped with a digital camera (Coolsnap-Pro; Photometrics).

### Fluorescent in situ hybridization

A previously described BAC probe was used to detect the PAR (Kauppi et al., 2011). The BAC (2.2 µg) was labelled with Biotin (Biotin-Nick Translation Kit, Roche). After alcohol precipitation in the presence of mouse Cot-1 DNA, the labeled DNA was resuspended in 25 µl formamide. Slides were first immunostained for CHD3/4 and SYCP3 as described above, and immunofluorescent images were captured at positions that were recorded. Then, the immunosignal was stripped from the slide as described (van de Werken et al 2013). 2.5 µl probe was used in a total volume of 20 µl hybridization mix. Denaturation was performed at 80°C, followed by hybridization overnight at 37°C, washings were performed in preheated formamid/1xSSC (1∶1 (V/V)) for 2×5 minutes with shaking, followed by 3 washes of 5 minutes in preheated 2xSSC, and one wash in 4xSSC/0.1% Tween-20 at room temperature. Detection of the biotin-labelled probe was performed with a primary antibody mouse anti-biotin (Roche diagnostics) and a secondary donkey anti-mouse Rhodamin (Jackson ImmunoResearch). Slides were mounted in Prolong gold with DAPI (Life Technologies), and images were captured at the recorded positions. The PAR probe reacts most strongly with the PAR of the Y chromosome and somewhat more weakly with that of the X. In addition, the PAR probe hybridizes to a few other chromosome ends that are also positive for CHD3/4. These additional foci are also observed when the CHD3/4 immuno is not performed, and therefore do not reflect a crossreaction of antibodies from the immune-staining with those used for detection of the FISH signal.

### Ethics statement

All animal experiments performed in Rotterdam were approved by the local animal experiments committee at Erasmus MC Rotterdam, The Netherlands (DEC Consult). The animals were housed in IVC cages under supervision of the Animal Welfare Officer. Any discomfort of animals was daily scored by the animal caretakers. No more than mild discomfort of animals was expected from the treatments, and no unexpected discomfort was observed. Experiments with mice performed in Germany followed the German Animal Protection Law (Tierschutzgesetz) and were approved by the experimental animal local authorities (Regierungspraesidium Giessen). Project reference number: V54-19c20/15cMR20/10 Nr. A 18/2010.

## Supporting Information

Figure S1 Expression of CHD3, CHD4 and CHD5 mRNA in 17 mouse tissues. Expression data was retrieved from www.ncbi.nlm.nih.gov/geoprofiles/ (dataset GD3052; expression values displayed on Y-axis are displayed as arbitrary units)). This dataset represents the analysis of 17 normal tissues from mouse and was obtained using the Affymetrix Mouse Genome 430 2.0 Array.
(TIF)
