# Supplementary figures and images for: Differential Expression and Sex Chromosome Association of CHD3/4 and CHD5 during Spermatogenesis
Source: PLoS One. 2014 May 21;9(5):e98203. doi: 10.1371/journal.pone.0098203 (PMC4029951; doi:10.1371/journal.pone.0098203)

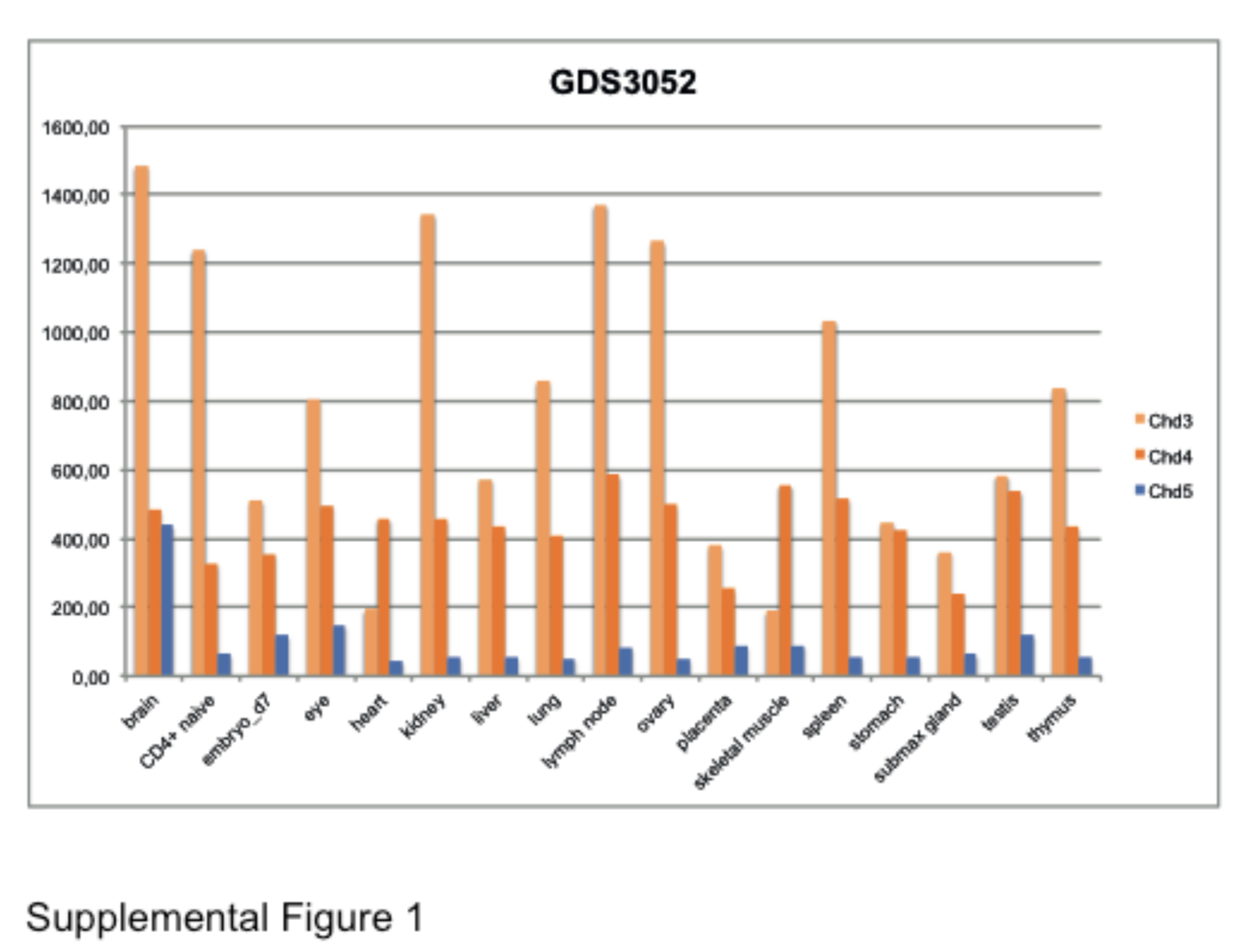

Supplement: Figure S1 — Expression of CHD3, CHD4 and CHD5 mRNA in 17 mouse tissues. Expression data was retrieved from www.ncbi.nlm.nih.gov/geoprofiles/ (dataset GD3052; expression values displayed on Y-axis are displayed as arbitrary units)). This dataset represents the analysis of 17 normal tissues from mouse and was obtained using the Affymetrix Mouse Genome 430 2.0 Array. (TIF) [file pone.0098203.s001.tif]
